# The ABC’s of Suicide Risk Assessment: Applying a Tripartite Approach to Individual Evaluations

**DOI:** 10.1371/journal.pone.0127442

**Published:** 2015-06-01

**Authors:** Keith M. Harris, Jia-Jia Syu, Owen D. Lello, Y. L. Eileen Chew, Christopher H. Willcox, Roger H. M. Ho

**Affiliations:** 1 School of Psychology, University of Queensland, St Lucia, Qld, Australia; 2 School of Public Health, University of Queensland, Herston, Qld, Australia; 3 School of Psychology, University of Newcastle, Newcastle, NSW, Australia; 4 Learning and Development Department, Illumina, Singapore, Singapore; 5 Hunter New England Mental Health, Newcastle, NSW, Australia; 6 Department of Psychological Medicine, National University of Singapore, Singapore, Singapore; University of Vienna, School of Psychology, AUSTRIA

## Abstract

There is considerable need for accurate suicide risk assessment for clinical, screening, and research purposes. This study applied the tripartite affect-behavior-cognition theory, the suicidal barometer model, classical test theory, and item response theory (IRT), to develop a brief self-report measure of suicide risk that is theoretically-grounded, reliable and valid. An initial survey (n = 359) employed an iterative process to an item pool, resulting in the six-item Suicidal Affect-Behavior-Cognition Scale (SABCS). Three additional studies tested the SABCS and a highly endorsed comparison measure. Studies included two online surveys (Ns = 1007, and 713), and one prospective clinical survey (n = 72; Time 2, n = 54). Factor analyses demonstrated SABCS construct validity through unidimensionality. Internal reliability was high (α = .86-.93, split-half = .90-.94)). The scale was predictive of future suicidal behaviors and suicidality (r = .68, .73, respectively), showed convergent validity, and the SABCS-4 demonstrated clinically relevant sensitivity to change. IRT analyses revealed the SABCS captured more information than the comparison measure, and better defined participants at low, moderate, and high risk. The SABCS is the first suicide risk measure to demonstrate no differential item functioning by sex, age, or ethnicity. In all comparisons, the SABCS showed incremental improvements over a highly endorsed scale through stronger predictive ability, reliability, and other properties. The SABCS is in the public domain, with this publication, and is suitable for clinical evaluations, public screening, and research.

## Introduction

Suicide continues to be a leading cause of death, touching the lives of people from every corner of the globe, and ranks as the 16^th^ leading cause of death [1]. Despite the seriousness and universality of this problem, instruments that evaluate and predict suicidality have not received the full attention they deserve. Demand is strong for empirically validated measures of personal risk for clinical and research efforts [2, 3]. However, many current instruments are inadequate for evaluation purposes [4]. Perhaps more than any other type of psychosocial assessment, suicide risk measures require focus on the minutiae of their psychometric properties and the validity of their outcomes. This study was aimed at producing incremental improvements in suicide risk evaluation by developing a brief self-report measure that incorporates theory and empirically evidenced suicidality attributes.

### Suicide Risk Assessment Models

It is imperative that the purpose of a test is clear and the instrument appropriate for the purpose [5, 6]. Suicide risk measures typically have two important goals, to assess both current suicidality and the potential for future suicidal behaviors. Currently, some clinicians choose not to use standardized suicide risk scales due to their overconfidence in clinical interviewing, and a perception that the instruments fail to capture essential aspects of suicidality [7]. However, an expert group concluded that clinicians are also unlikely to assess the suicidal person’s inner state, their subjective experience of being suicidal [8]. Risk assessment models can help guide and encourage professional evaluations.

The tripartite model postulates that an attitude (e.g., toward suicide or death) is comprised of three correlated but distinct components: affect, behavior, and cognition [9]. The ABC model encompasses common suicidality factors, which might be useful for assessing suicide risk [10]. Kral and Sakinofsky [11] proposed a two-tier clinical assessment model that includes sociodemographic factors to understand the client’s general risk level, and subjective factors (thoughts, emotions, suicidal history) to identify individual risk, but has been inadequately tested. Incorporating demographic factors may, however, be counterproductive for standardized individual assessment [12]. Many suicide risk measures, such as the SAD PERSONS [13] and the Manchester Self Harm Rule (MSHR) [MSHR; 14], use dichotomous items on demographics and select risk and protective factors. However, these indexes have been criticized for inaccurate risk classifications, which can lead to a drain on psychiatric services [15, 16].

Jobes’ Suicide Status Form (SSF) [17] is a clinician-administered measure stemming from the theoretical works of Shneidman (psychological pain, agitation) [18], Beck (hopelessness) [19], and Baumeister (self-hate) [20]. It includes items on suicidal affect, behaviors, and cognition. The SSF is the most likely candidate for a gold standard in clinical evaluation. Unfortunately, its’ length and inclusion of qualitative responses make it inconvenient for some screening and research applications. Following on Shneidman’s [18] depiction of suicidality as a “storm in the mind,” and ABC theory, the suicidal barometer model (SBM) was recently introduced to guide risk evaluations. The SBM is based on theory and empirical evidence that suicidality is a volatile state, with strong implications for both current and future personal risk [11, 21, 22]. The SBM proposes that risk measures should capture the individual’s experience of that internal storm, through subjective behavioral intentions, life-death affect, and suicidal cognition.

### Suicidal Affect, Behavior, and Cognition

Affect is the most ambiguous factor of the tripartite model and the least common for suicidality assessment. Several terms have been used that might be regarded as death or suicide-related affect. They include the wish to live (WTL) and wish to die (WTD), which Kovacs and Beck referred to as a “motivational dimension” [23], emotions [21], and passive ideation [24]. Hopelessness is an affect with strong associations to suicidality. The Beck Hopelessness Scale (BHS) has proven useful in research and risk assessment, and might be a unique suicidal attribute [19, 25]. There is, however, some contradictory evidence on relationships between affect and suicidality. A study of patients presenting with suicidal symptoms found inconsistent associations between affect and suicide attempt status [26], while a large longitudinal study determined the combination of lifetime cognition and death-related affect were the best predictors of suicide death [24]. WTL and WTD items have proven useful in assessing suicidality, and are included in Beck’s Scale of Suicidal Ideation (SSI) [27], and the SSF [28]. Overall, there is strong evidence that suicidal affects can be valid indicators of current and future risk.

A considerable body of empirical evidence demonstrates suicidal behaviors, such as plans and attempts, can be predictive of suicide [29–31]. Of importance to risk assessment, research has shown that including the individual’s intent to die improves the validity of past suicidal behaviors as indicators of current and future risk [32, 33]. Many instruments, such as the Suicidal Behaviors Questionnaire-Revised (SBQ-R) [34] and the Suicide Intent Scale (SIS) [35], include items on communication of suicidality. However, Kovacs et al. [36] concluded, from an examination of US suicide attempters, that prior verbalization of suicidality had little relationship with WTD during the attempt, and may be a manifestation of personal style. More recently, a large study of French university students found higher risk suicide attempts included less communication of suicidality [37], while a psychological autopsy study of 200 Chinese suicide victims revealed about 60% had not communicated their suicidality, in any way, prior to death [38]. Non-suicidal self-harm (NSSH) is also included in some suicide risk measures, such as the Self-Injurious Thoughts and Behaviors Interview [39]. However, recent research found that including NSSH did not provide additional predictive ability to a model including suicidal cognition and behaviors [29]. Overall, there is considerable evidence that past suicidal plans and attempts should be considered for evaluation of current and future risk, but other behaviors, such as NSSH and communications, may not be valid factors for many individuals.

Suicidal cognition, or ideation, is considered to be a defining attribute of suicidality [8, 40, 41]. Numerous studies have provided empirical evidence demonstrating the importance of suicidal cognition for current and future suicide risk [24, 29, 42]. Other than select instruments, such as the MSHR and SAD PERSONS, most suicide risk measures include at least one suicidal cognition item.

Scales require relevant definitions of the construct to enable effective assessment [5, 6]. Many definitions of suicidality are strictly behavioral. The suicidal mind, the extremely distressing experience of the suicidal individual, is often absent. For example, one expert group defined suicidality as “completed suicide, suicide attempt, or preparatory acts toward imminent suicidal behavior” [43]. Some have even argued that the term suicidality be abandoned, with focus on specific suicidal behaviors [44]. Others propose better representation of the lived experience of being suicidal [7, 8]. For example, Shneidman described suicide as an “extreme (unbearable) psychological pain coupled with the idea that death (cessation) can provide a solution to the problem of seemingly unacceptable mental distress” [18]. For this study, we define suicidality as current suicide-related distress (which may include affective, behavioral, and cognitive attributes), with potential for future suicidal distress and behaviors.

### Best Practice Scale Properties

There are numerous factors to consider when developing or testing a measure. Unfortunately, those minutiae, forming the structure of the instrument, are often ignored in suicide risk assessment. Here, we summarize relevant findings and recommendations of psychometricians and scale development experts. Single-item measures of a construct, including suicidality as assessed in the Beck Depression Inventory (BDI II) [45] and the Patient Health Questionnaire (PHQ-9) [46], should be avoided as there are only rare situations when single items perform as well as validated multiple-item measures [5, 47]. Dichotomizing items (e.g., yes/no) or outcomes (e.g., suicidal/nonsuicidal), reduces validity by constraining the amount of information that can be captured on the latent trait, and should also be avoided whenever possible [48–50]. Some psychometricians have determined the ideal number of item response choices to be 4–7 [51, 52]. Fortunately, item response theory (IRT) analyses can help verify response format validity [48, 53]. Psychometric study has also shown that verbally labeled responses (e.g., poor, fair, good) differed by 0.7 to 1.3 points, rather than the equidistant 1.0 used for item scoring [54]. An advantage of verbally labeling only anchor points is obtaining interval level data, through equidistant response categories. Another important consideration is item weighting. IRT analyses can determine whether items make equal or disproportionate contributions to scale totals [48, 53].

Differential item functioning (DIF), or item bias, refers to a situation when respondents with the same trait level, but belonging to different groups, show dissimilar probability distributions on responses to a particular item [55]. IRT analyses have found DIF for white and Asian Americans on depressive symptoms [56], and for age groups on the BDI [57]. Similarly, classical test theory (CTT) analyses found lower internal reliability for Asian American university students, compared with white students, on the Positive and Negative Suicide Ideation inventory [58]. Those findings indicate that the measures do not function the same for some groups. DIF checks have yet to be applied to suicide risk assessment, although they are important procedures for test development and checking inter-group validity [59].

For scale development, representativeness on the target constructs does not require random sampling from target populations, it requires samples where relationships among items, or constructs, are the same as in target populations [5]. A recent study found a large university community sample reported lower ranges of high-risk mental health symptoms and substance use, and lower scale reliability, compared with an online community sample [60]. Another possible obstacle to response validity is social desirability bias [61, 62]. However, that can be significantly reduced, and self-disclosure of personal information increased, through anonymous assessment methods [62–64]. Online surveys may be particularly useful for examining suicidal individuals, as they have been shown to be more active online than nonsuicidal people [65, 66]. Those findings point to advantages of anonymous surveys and a possible weakness of university samples when developing measures of stigmatized constructs, such as suicidality. Given the empirical evidence for these fundamental scale development practices, the burden of proof is on test developers and administrators to justify variations, such as including dichotomous items or outcomes, or developing scales with only university students.

### Current Measures of Suicidality/Suicide Risk

While a full review of the numerous suicide risk measures is beyond the scope of this study, there are popular and recommended instruments that deserve consideration. Test administrators are likely to refer to expert recommendations to choose the best available measure for their purposes. However, expert opinions can be based on a variety of standards. In Range and Knott’s [21] earlier review of 20 suicide risk instruments, scales were judged to assess the theoretically important factors: emotion, behavior, and cognition. The authors determined that only 30% of those scales assessed an emotional component of suicide risk, only 25% at least two factors, while no instrument was judged to assess all three attributes. Based on reported reliability, validity, and theoretical grounding, they recommended the SSI, Linehan’s Reasons for Living Inventory, and the SBQ-R. The American Psychiatric Association [67] did not recommend any specific tool, but highlighted the SSI and SBQ-R as valuable in assisting clinical judgment. An expert panel in New Zealand recommended only the BHS, stating it “has the best generic application for screening for suicide risk amongst adults, adolescents, inpatients, outpatients and people seeking assistance from emergency departments” [68]. However, earlier research determined the SSI-W to be more effective than the BHS for assessing suicide risk [69]. The British Medical Journal, as part of their best practices initiative, recommended the Tool for Assessment of Suicide Risk (TASR), stating that it “helps to ensure that the most important issues pertaining to suicide risk are considered” [70]. The TASR [71] consists of dichotomous items on affect, behaviors, and cognition, as well as demographic factors (e.g., age, sex), medical illness, and reasons for living. The scale developers provided no psychometric properties of the instrument, nor any indication of its validity in assessing suicide risk.

The SBQ-R and C-SSRS were two of four measures endorsed by the US Substance Abuse and Mental Health Services Administration’s Center for Integrated Health Solutions [72]. The initial study of the C-SSRS reported high internal reliability for a small sample (α = .95, *N* = 124), but low reliability with a larger sample (α = .73, *N* = 549) for one of four subscales, while others were not evaluated [73]. The C-SSRS consists of clinician-administered prompts with mostly dichotomous scoring options [74]. It includes cognition, behaviors, and one dichotomous item on affect. An electronic version (eC-SSRS) consists of ‘electronic’ clinician-administered dichotomous items, and demonstrated some predictive ability, but rather low sensitivity and specificity rates [75]. It is notable that few measures assess all three ABC attributes. The SSI includes items on cognition and affect, but behaviors are limited to current suicide planning and communications. The SIS includes items on suicidal affect and behaviors (regarding a recent attempt), but the one cognition item assesses impulsiveness of an attempt. The Adult Suicide Ideation Questionnaire [76] includes items on cognition and affect, but the behavior items are limited to suicidal communications. Nearly all of these instruments require fees for use. Currently, there are no known self-report public domain measures that include all ABC attributes.

### Study Aims

This study was aimed at building on the pioneering suicide risk evaluation work of Shneidman [18, 41], Beck and Kovacs [23, 27], Osman and Gutierrez [34, 77], Linehan [61, 78], Jobes [7, 79], and many others. Our goal was to create a brief self-report measure of suicidality/suicide risk that makes an incremental improvement over an existing standard. After excluding pay per use and clinician-administered measures, the scale that best met criteria for a reliable and valid self-report measure, and which has been endorsed by numerous experts and professional organizations [21, 40, 72], was the SBQ-R. We therefore included the SBQ-R as a comparison measure. We hypothesized that a new scale could demonstrate construct validity (unidimensionality), sensitivity to change, higher reliability, statistically greater predictive ability (stronger associations with future suicidal behaviors and suicidality), greater convergent validity (stronger associations with suicide risk and protective factors), and would be more effective at capturing information relevant to low, moderate, and high suicidality.

## Method

### Ethics Statement

All participants were anonymous volunteers, and were informed of their rights to not respond to any items, or to withdraw at any point. The studies were approved by the University of Queensland Human Research Ethics Committee (HREC 05PSYCHPHD67VS); JCU (H3841); the University of Newcastle (H20120299; and Hunter New England Health (HREC13HNE235). Informed consent was written (online via agreeing to the appropriate informed consent form).

### Participants

Data came from four independent samples. Study 1 (*N* = 359) participants were 195 online and community volunteers, and 164 university students in Singapore, aged 18 to 72 years (*M* = 27.88, *SD* = 11.14); 77.8% women; 58.2% Asian, and 41.8% white. Study 2 included 1007 online survey participants, 62.3% women; aged 18–71 years (*M* = 30.37, *SD* = 10.54); 82.2% white. Study 3 included 713 online survey participants, 77.1% women; aged 18–71 years (*M* = 31.48, *SD* = 13.53); 78.5% white. Study 4 included 72 patients, out of a possible 81 (89% response rate) who were at various stages of a one-year Dialectical Behavior Therapy (DBT) program for borderline personality disorder (BPD) in Australia. They were clinically evaluated as suicide-risk; 86.1% women; aged 18–55 years (*M* = 26.96, *SD* = 8.89); 94.4% white. To test predictive validity, Study 4 also included 54, out of a possible 63 (86% response rate), participants assessed a second time (T2). The remaining 18 patients had completed or stopped treatment and were unavailable for follow-up assessment. The full study consisted of 2,151 participants; 70.6% women; aged 18–76 (*M* = 30.27, *SD* = 11.79); 71.1% white, 19.1% Asian, and 9.8% other ethnicities. Ethnicity was determined by self-report. Due to small numbers, those indicating ethnicities other than white or Asian were grouped as other. Studies 1–2 included university undergraduates (42.1% and 1.7%, respectively) earning partial course credit, some Study 1 community members received a Suicide Study Group t-shirt, and Study 4 participants received a small gift, such as a stress-release squeeze ball. No incentives were offered to other participants.

### Measures

#### Suicidal Behaviors Questionnaire-Revised [34]

The SBQ-R’s four items are scored as follows: history of suicidal Behaviors (1 = “never,” 6 = “I have attempted to kill myself, and really hoped to die”); past year suicidal Ideation (1 = “never,” 5 = “very often”); Communication of suicidality (1 = “no,” 5 = “yes, more than once, and really wanted to do it”); and Prediction of future suicide attempts (0 = “never,” 6 = “very likely”). Ideation responses included “sometimes (2 times)” and “often (3–4 times).” Prediction included “no chance at all,” and “rather unlikely.” As those labels lacked face validity for equidistant response points, expert recommendations were followed and only anchors were verbally labeled [54]. Osman et al. [34] revised the scoring of Behaviors and Communications for total scores, with higher totals indicating greater suicidality.

#### Psychosocial Measures

Studies 1–3 included the following measures of suicide risk and protective factors: depressive symptoms, Center for Epidemiologic Studies-Depression scale (CES-D, Study 1) [80], CES-D 10 (Study 2) [81], Depression Anxiety and Stress Scales (DASS, Study 3) [82]; stress and anxiety (DASS, Study 3); a 5-item version of the Beck Hopelessness Scale (BHS5) [83]; a 5-item version of the UCLA Loneliness Scale (UCLA5) [84]; the Multidimensional Scale of Perceived Social Support (MSPSS, Studies 1–3) [85]; and satisfaction with life (SWL, Study 3) [86]. We included weekly hours of online shopping (Study 2) [66], and the International Personality Item Pool (IPIP) Intellect scale (Study 3) [87], as discriminant validity checks [88]. All measures demonstrated adequate internal reliability (α ≥ .80).

### Procedure

Study 1 was a scale development project that included focus group discussions, piloting of test items, and a survey that included the test item pool and measures of related constructs. Studies 2–4 were secondary analyses of the suicide risk scales, and measures of related constructs. Study 1 included an anonymous online survey and anonymous computer-administered survey participants in a university computer laboratory. Studies 2 and 3 were anonymous online surveys. Studies 1–3 were promoted through online postings which informed participants that the survey examined suicidality and other variables, as well as snowballing. These were purposive surveys, with oversampling of suicide-risk individuals to better examine properties of study variables. Each study was promoted separately, and were not concurrent. To ensure strict anonymity, participant IP addresses were not collected. While that allows for the possibility of repeat participation, examination of response characteristics showed no evidence of such. Participants first indicated their consent to participate in a study on suicide and other factors and that they were aged 18+ years. They were next asked to complete various psychosocial measures and demographic items. Only the consent item was mandatory. Whenever a participant ended a survey they were taken to an exit page, with links and phone numbers of free crisis support. Study 4 patients completed anonymous pen-and-paper surveys during DBT therapy breaks. Their T2 assessments occurred 7–11 weeks later, after they completed a DBT module. That provided sufficient time to avoid recall effects and to examine changes in suicidality. Due to the high risk nature of this group, great care was made to reduce the burden on these participants. Therefore, the T2 assessments were limited to select questions. The surveys also included additional measures beyond the scope of this study.

### Analyses

Data cleansing included identification and treatment of univariate and multivariate outliers, and missing values [89]. Missing values were shown to be missing completely at random and were replaced through the expectation maximization procedure. IRT analyses do not assume the same data characteristics as many CTT tests, such as item skew or a normal distribution, but do require item sets to be unidimensional, and that there not be an additional latent trait that explains person-item characteristics [48, 53]. IRT models should be chosen first according to the data characteristics, then verified through theoretical and statistical checks [48]. The scales include polytomous items with varying response formats and meanings, making the graded response model (GRM) most suitable, particularly as the items may vary in their ability to capture information on the latent trait [90]. GRM requires response options to be ordered, i.e., a given item’s response choice captures a higher level of theta (latent trait) than any preceding response on that item. Therefore, Likert-type responses are suitable, but categorical items, such as Behaviors, may or may not be ordered. Concurrent study determined that the SBQ-R’s scoring of Behaviors was not valid, and resulted in a new ordering used in this study. IRT analyses require large sample sizes, with a minimum of 500 recommended for GRM [91]. As studies 1 and 4 used different response ranges for WTL and WTD, we combined data from studies 2 and 3 (*n* = 1,720) for the following analyses. Most analyses used SPSS v. 22. For IRT analyses we used R 3.1.2 (Pumpkin Helmet), ltm package [92]; and EasyDIF for DIF analyses [93].

#### Reliability

For testing internal reliability, we were guided by expert opinion recognizing α ≥ .80 as adequate for research, and .90–.95 as preferable for clinical purposes [5, 6, 94]. Very high alphas are concerning as they may indicate item redundancy [95]. We included Spearman-Brown prophesy (split-half) coefficients as important psychometric data is missing when reporting only α for internal reliability [96].

#### Validity

We followed recommendations by employing an iterative process involving exploratory factor analyses (EFA) to determine which items showed strong loadings on common factors [89, 97, 98]. EFAs also tested construct validity, i.e., unidimensionality of the latent trait. Pearson correlations tested convergent and predictive validities of the items and scales [99, 100]. Steiger’s z scores were calculated to test whether the new measure showed statistically stronger correlations than the comparison measure on related factors. Sensitivity to change was assessed through smallest real difference (SRD) calculations [101]. IRT analyses were used to assess item and scale abilities to capture information on low, moderate, and high levels of theta. DIF analyses evaluated items by sex, age group, and ethnicity.

#### Item pool

Following scale development guidelines [5, 102], we selected items broadly related to suicide risk from previously validated measures and the suicidology literature: e.g., the SSF [28], the BHS [83], the SSI [27], WTL and WTD [23], an internal suicidal Debate [103], history of suicidal Behaviors, and revised SBQ-R items. The reviewing committee (n = 14) included clinical psychologists, a PhD suicidology expert, and clinical and 4^th^-year psychology students who completed suicidology coursework. Discussions on face validity, theory, and item clarity, resulted in a 43-item pool. We also revised items to remove any jargon and improve semantic compatibility [104]. Of note, we modified the SSI item Future to “I accept the possibility of possibly killing myself.” Non-anchor response categories were not verbally labeled (excluding Behaviors).

## Results

Initial analyses revealed a lack of linearity between some items. Those items were removed, and included “I would try to protect my life if I found myself in a life-threatening situation.” The remaining 17 items met requirements for EFA (KMO > .80, 21 cases/variable) [89]. We chose the maximization likelihood extraction method with oblique rotation, as we expected multiple factors, if they exist, to be correlated [97]. Costello and Osborne recommend at least five items loading ≥ .50 to identify a strong factor; and only retaining items with communalities (h^2^) ≥ .40, otherwise they do not relate strongly to others. Items not meeting these criteria were removed one by one, starting with the worst fitting item.

EFA revealed two factors, which were comprised of seven items, and three BHS items. We removed the BHS items as the study was aimed at producing a unidimensional scale specific to suicidality. For the remaining seven-items, FA results showed Future had slightly lower communalities and factor loadings than the similar Prediction item. We also examined GRM results, which showed that Prediction had a higher information function (IF), a = 2.46, than did Future, a = 2.16. As they are similar, we retained Prediction as the sounder of the two.

### Suicidal Affect-Behavior-Cognition Scale (SABCS, Appendix)

The resulting six-item SABCS relates well to ABC theory. It includes items on death-related affect, WTL and WTD; suicidal Behaviors; suicidal cognition, Debate and Ideation; and Prediction of future suicide attempts, a self-assessment item which may have underlying cognitive and affective attributes. A briefer version, the SABCS-4, consists of WTL, WTD, Debate, and Behaviors. However, the full version is strongly recommended. Items are totaled, with higher scores indicating greater suicidality.

### Scale Reliability

Table 1 shows the SABCS demonstrated high internal reliability, but showed no evidence of item redundancy (i.e., inter-item r ≥ .90) [89]. For T2, we modified Debate and Behaviors to capture past two-week levels of those constructs, and used the SABCS-4, which also showed high internal reliability. To test the validity of the item response ranges for WTL and WTD, which have been used with 2–10 category response formats in the past, we varied response ranges from 5, 7, and 10 levels. All response options for the six SABCS items were endorsed in Studies 1–3, providing a degree of validity for the response ranges used [5]. That included 5 and 10-level response formats of WTL and WTD. For Study 4, however, the lowest response choice for Ideation and two lowest choices for Behaviors were not endorsed by any of the clinical participants. That is likely a reflection of their high-risk status, resulting in lower item correlations [89].

**Table 1:** Psychometric properties of the Suicidal ABC Scale and the Suicidal Behaviors Questionnaire-Revised.

Scale/study	Range^†^	M	SD	Inter-item r	Item-total r	α	S-B	SE_m_
SABCS								
Study1	7–34	12.03	7.01	.46–.79	.69–.86	**.91**	.91	2.10
Study 2	5–44	11.01	6.87	.54–.82	.74–.82	**.91**	.92	1.94
Study 3	5–44	14.82	9.06	.58–.79	.75–.85	**.93**	.94	2.22
Study 4	12–38	26.04	7.38	.19–.82	.33–.81	**.86**	.90	2.34
SABCS-4								
Study 4 T2	2–24	10.91	6.70	.67–.85	.81–.87	**.92**	.91	1.90
SBQ-R								
Study 1	3–18	6.92	3.84	.45–.68	.55–.75	**.80**	.78	1.72
Study 2	3–18	6.60	3.84	.52–.71	.60–.78	**.83**	.85	1.58
Study 3	3–18	8.31	4.72	.50–.77	.57–.82	**.83**	.84	1.95
Study 4	6–18	13.58	3.22	.10–.66	.25–.61	**.59**	.47	2.06

Study 1, n = 359; Study 2, n = 1007; Study 3, n = 713; Study 4, clinical sample, n = 72; SABCS-4 = 4-item SABCS; T2 = Time 2, n = 54. α = Cronbach’s α; S-B = Spearman-Brown prophecy coefficient; SE_m_ = standard error of measurement.

^†^Studies used different response ranges for some SABCS items.

### Construct Validity

For the final FA with Study 1, parallel analysis, the scree plot, and the lenient eigenvalue > 1.0 rule, indicated a single-factor solution explaining 70.8% of the variance in the latent trait. Table 2 shows all items loaded strongly on one factor, surpassing the .50 standard, and meeting Comrey and Lee’s [105] highest criteria of “excellent” factor loadings (≥ .71). Communalities were moderately high (mean h^2^ > .65), providing some confidence that the observed structure is likely to be a good representation of population factors [102, 106]. Confirmatory factor analysis (CFA) is commonly used to verify a scale’s factor structure in follow-up studies. However, some experts suggest additional EFAs as a more conservative test [107]. Unlike CFAs, EFAs can provide evidence that there is no superior factor structure with the new data. Table 2 shows a single factor solution explained between 61.7% (clinical sample) to 76.6% of the variance in the underlying trait. All loadings of Studies 1–3 met Comrey and Lee‘s [105] “excellent” criteria. However, for Study 4, Ideation met the “very good” criteria, and Behaviors only met their “poor” criteria (≥ .32), but also met minimum standards of other experts [89, 108]. There were at least five items with *h* ^2^ ≥ .50 for all studies. However, the Behaviors item showed low *h* ^2^ for Study 4, likely due to participants not endorsing the lowest response choices [89]. EFAs confirmed SABCS unidimensionality, satisfying a primary IRT requirement [53].

**Table 2:** Factor loadings and communalities of Suicidal ABC Scale items.

	Factor loadings				Communalities (h^2^)			
Item	S1	S2	S3	S4	S1	S2	S3	S4
Ideation	.86	.86	.88	.71	.68	.68	.73	.54
WTD	.80	.81	.86	.85	.71	.70	.74	.73
Prediction	.80	.82	.88	.87	.60	.62	.72	.68
WTLr	.72	.86	.80	.81	.61	.75	.66	.70
Debate	.90	.75	.84	.73	.78	.58	.69	.58
Behaviors	.74	.79	.80	.34	.60	.63	.64	.18
1^st^ eigenvalue	4.25	4.40	4.60	3.70				
2^nd^ eigenvalue	0.71	0.58	0.51	0.93				
Variance	70.8%	73.4%	76.6%	61.7%				

S1 = Study 1 (n = 359), S2 = Study 2 (n = 1007), S3 = Study 3 (n = 713), S4 = Study 4 (n = 72). Ideation = suicidal ideation, WTD = wish to die, Prediction = prediction of future suicide attempts, WTLr = wish to live reverse-scored, Debate = internal suicidal debate, Behaviors = history of suicidal behaviors, Variance = percentage of total trait variance explained by the retained factor.

### Predictive Validity

T2 analyses of BPD patients were necessarily brief, to reduce the burden on these high-risk patients, which resulted in the omission of two SABCS items (Ideation and Prediction), for the four-item SABCS-4. As shown in Table 1, the SABCS-4 demonstrated sound psychometric properties. For the prospective analyses of Study 4, we considered Behaviors (which includes suicidal plans and attempts), and overall suicidality (SABCS-4 total), to be the primary outcome variables. Within two weeks prior to T2 assessment, two participants (3.7%) reported suicide attempts with non-lethal intent, one (1.9%) reported an attempt with lethal intent, and 12 participants (22.2%) reported suicide plans with intent to die. There were no suicide deaths. Table 3 shows WTD and WTL were the best single-item predictors of Behaviors and total suicidality. Debate, Prediction and Ideation were also strong predictors of T2 outcomes, while Behaviors was a surprisingly weak predictor of T2 outcomes. Communications showed no significant predictive validity. Steiger’s z scores demonstrated the SABCS was, as hypothesized, a stronger predictor of T2 outcomes than the SBQ-R (ps < .01). To summarize the predictive abilities of the two measures, R^2^ values show the SABCS explained 46% of the variance in T2 suicidal behaviors, and 53% of total T2 suicidality. The SBQ-R explained 21% of the variance in T2 behaviors and 25% of T2 suicidality. We next examined the sensitivity to change of the SABCS-4 (as the full SABCS was not used for T2). Using 95% CI of SE_m_, SRD = 5.27, which was surpassed by 42.6% of the clinical participants at T2, demonstrating the scale is adequately sensitive to meaningful changes in suicidality.

**Table 3:** Pearson correlations between Time 1 and Time 2 suicidality measures of a clinical sample.

Time 1 predictor	Time 2 (n = 54)				
	Behaviors (2-week)	WTD (current)	WTL (current)	Debate (2-week)	SABCS-4
WTD (current)	.65***	.66***	-.57***	.61***	.69***
WTL (current)	-.62***	-.62***	.66***	-.65***	-.70***
Debate (lifetime)	.55***	.54***	-.40**	.55***	.57***
Predict (current)	.51***	.56***	-.51***	.53***	.58***
Ideation (past year)	.47***	.51***	-.37**	.50***	.52***
Behaviors (lifetime)	.33*	.29*	-.16	.23	.27*
Comm. (lifetime)	-.08	-.04	.21	-.09	-.11
**SABCS total**	**.68** ***	**.69** ***	**-.59** ***	**.67** ***	**.73** ***
SBQ-R total	.46***	.51***	-.37**	.46***	.50***
Steiger’s z	3.71***	3.12**	3.43***	3.52***	4.09***

Time 2 was 7–11 weeks after Time 1. WTD = wish to die; WTL = wish to live; Debate = internal suicidal debate; Predict = prediction of future suicide attempts; Ideation = suicidal ideation; Behaviors = suicidal behaviors; Comm. = communication of suicidality; SBQ-R = Suicidal Behaviors Questionnaire-Revised; SABCS = Suicidal ABC Scale; SABCS-4 = total of T2 items. Steiger’s *z* compared SABCS and SBQ-R correlations.

*p < .05

**p < .01

***p < .001.

### Convergent Validity

We next conducted Pearson correlations of the SABCS and SBQ-R with measures of suicide risk and protective factors, for Studies 1–3. Table 4 shows evidence of convergent validity as all SABCS correlations were statistically significant and in expected directions. Evidence of discriminant validity was shown through a non-statistically significant association with online shopping, and a very small, but statistically significant, correlation with Intellect. Steiger’s *z* scores showed the SABCS, as hypothesized, demonstrated statistically stronger convergent validity than the SBQ-R on all comparisons.

**Table 4:** Pearson correlations of psychosocial factors with the Suicidal ABC Scale and the Suicidal Behaviors Questionnaire-Revised.

Variable (sample, measure)	SABCS	SBQ-R	Steiger’s z
Depression (S1, CES-D)	.74	.68	5.53***
Depression (S2, CES-D 10)	.67	.61	7.15***
Depression (S3, DASS)	.80	.75	5.94***
Hopelessness (S1, BHS5)	.71	.64	5.99***
Stress (S3, DASS)	.66	.63	2.85**
Anxiety (S3, DASS)	.66	.63	2.85**
Loneliness (S1, UCLA5)	.57	.51	4.47***
Satisfaction with life (S3, SWL)	-.69	-.67	1.97*
Social support (S1, MSPSS)	-.48	-.42	4.25***
Social support (S2, MSPSS)	-.61	-.55	6.70***
Social support (S3, MSPSS)	-.66	-.62	3.76***
Online shopping (S2, hours)	.02	.02	0.00
Intellect personality (S3, IPIP)	-.12	-.09	2.24*

S1 = Study 1 (n = 359), S2 = Study 2 (n = 1007), S3 = Study 3 (n = 713). CES-D = Center for Epidemiologic Studies-Depression scale; DASS = Depression Anxiety Stress Scales; BHS5 = 5-item Beck Hopelessness Scale; UCLA5 = 5-item UCLA Loneliness Scale; MSPSS = Multidimensional Scale of Perceived Social Support; IPIP = International Personality Item Pool. Correlations between SABCS and SBQ-R with other variables were statistically significant, ps < .05, excluding online shopping.

*p < .05

**p < .01

***p < .001

### Item Response Theory Analyses

We first tested whether a constrained GRM model, where all items are given equal weighting, or an unconstrained model, where items are allowed to vary in their ability to capture the latent trait, better fit the data. Results showed that the unconstrained model was a better fit, LRT = 26.19 (df = 5), p < .001. We therefore used unconstrained GRM for the following analyses. For GRM, item discrimination levels (a), or item slopes, indicate the peak level of theta an item discriminates on; b parameters show the range of difficulty levels of an item, the item’s ability to discriminate at low and high levels of theta [53]. As shown in Table 5, all SABCS items had relatively high slopes (a > 2.0), and showed a greater range of b parameters than the SBQ-R. WTD and WTLr were the most difficult of the SABCS items (b > 2.0), meaning, participants who score high on these items, compared to others, are most likely to be at high risk. The amount of information an item provides depends on both the size of slope (a), and the spread of the b thresholds [53, 109]. The sum of the item IFs provides the test IF. As predicted, the SABCS captured substantially more total information on theta than the SBQ-R. The relative efficiency of the SABCS to the SBQ-R = 1.94 (i.e., 51.80/26.77), showing the SABCS functions as if it were 94% longer. At an item level, WTD explained more information on theta than did other SABCS items, which calls into question the validity of equal item weighting.

**Table 5:** Graded response model analyses of the Suicidal ABC Scale and the Suicidal Behaviors Questionnaire-Revised.

Scale/item	a	b_L_	b_U_	IF	Pct.
SABCS				51.80	
WTD	3.60	0.46	2.35	11.51	22.2%
WTLr	3.01	0.23	2.44	8.72	16.8%
Ideation	3.67	-0.00	1.26	8.57	16.6%
Prediction	2.68	-0.27	2.11	7.95	15.3%
Debate	2.73	-0.64	1.66	7.60	14.7%
Behaviors	2.79	-0.69	1.65	7.45	14.4%
SBQ-R				26.77	
Ideation	4.11	-0.04	1.09	9.95	37.1%
Behaviors	3.00	-0.72	1.21	7.52	28.1%
Prediction	2.43	-0.28	1.95	6.59	24.7%
Comm.	1.78	0.62	1.64	2.71	10.1%

N = 1,720; WTD = wish to die; WTLr = wish to live reverse-scored; Ideation = suicidal ideation; Prediction = prediction of suicide attempts; Debate = internal suicidal debate; Behaviors = history of suicidal behaviors (SABCS uses SBM scoring; SBQ-R uses Osman et al. scoring [34]); Comm. = communication of suicidality; a = item discrimination level; b_L_ = lowest item difficulty threshold; b_U_ = upper item difficulty threshold; IF = information function; Pct. = percentage of total scale information.

**Fig 1:**
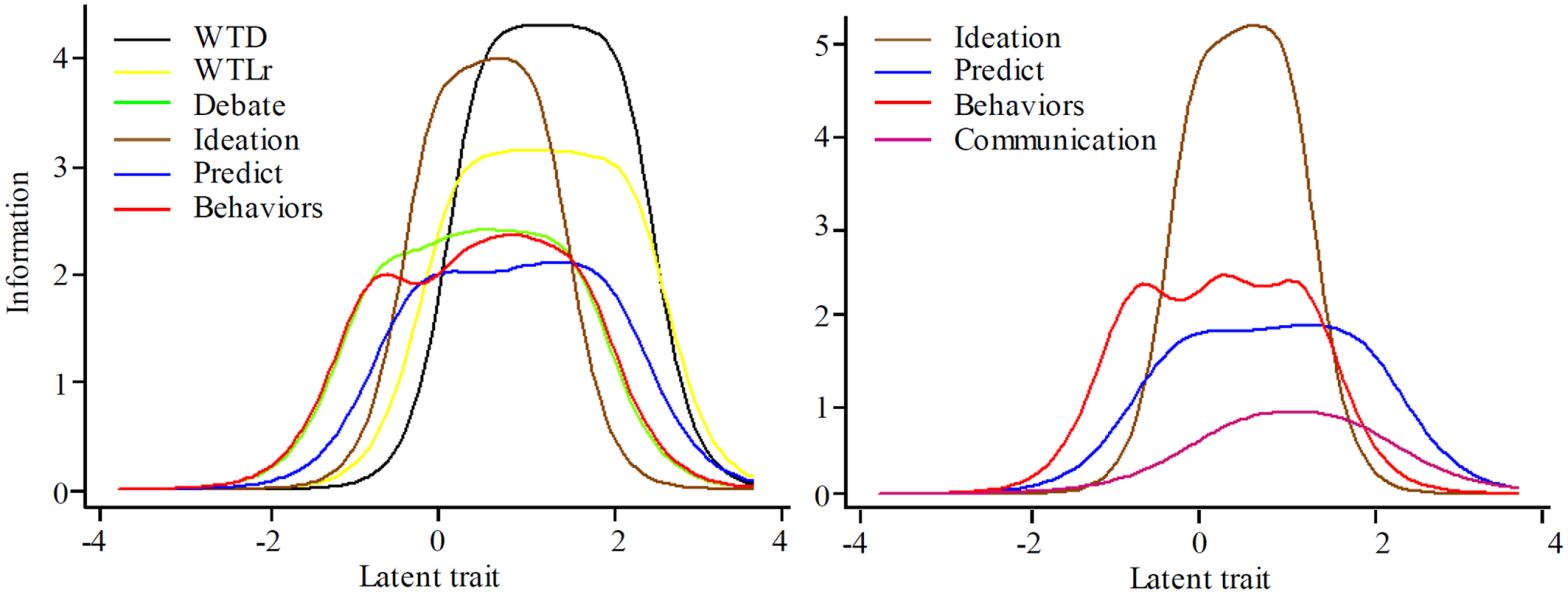
Item information curves: A = Suicidal ABC Scale, B = Suicidal Behaviors Questionnaire-Revised.

Fig 1A and 1B show the item information curves of the two scales. Fig 1A shows Debate and Behaviors were best at capturing lower levels of theta, WTD and Ideation were best at capturing middle to high levels, and WTLr was best at capturing very high levels of theta. Fig 1 shows SABCS items capture more information at each level, but particularly on the highest range of theta (2–4 on the x-axis) than SBQ-R items. Interestingly, although they assess different suicidal attributes (i.e., behavior and cognition), the Behavior and Debate items show very similar patterns for capturing information on theta. This, however, does not demonstrate item redundancy, but rather similar abilities for measuring the latent trait. We also examined the item category characteristic curves (CCCs), including for different response ranges of WTL and WTD (i.e., 5/7/10 points). Results showed ten response points is probably too many, and that seven response points may be too many for other items. We therefore suggest response ranges of 6–7, based on the CCC plots and the GRM results shown in Table 5 (see Appendix).

DIF analyses compared response patterns by age groups (18–29, 30–49, and 50–76 years), ethnicity (white, Asian, and other), and by sex. We used the K Index to test DIF, as it uses GRM and allows for non-dichotomous groupings [93]. Kumagai demonstrated that a significant K Index is comparable to a Mantel-Haenszel chi-square statistic Δ > 1.5, indicating a large DIF [110]. Analyses revealed no evidence of DIF for any items, meaning they functioned similarly on the latent trait across these groupings.

## Discussion

Through multi-method, multi-study, construct validation, the newly developed Suicidal Affect-Behavior-Cognition Scale demonstrated incremental improvements over an existing standard in self-report suicide risk evaluation. The SABCS showed stronger abilities for predicting future suicidal behaviors and suicidality, convergent validity, internal reliability, and sensitivity to change. Importantly, no items showed evidence of DIF. Factor analyses confirmed unidimensionality and construct validity, while hopelessness was shown to be a separate but related factor. The six-item SABCS captures affective, behavioral, and cognitive attributes of suicidality. That implies that affect, behaviors, and cognition are not independent factors, but are unique attributes of this unidimensional but complex trait.

**Fig 2:**
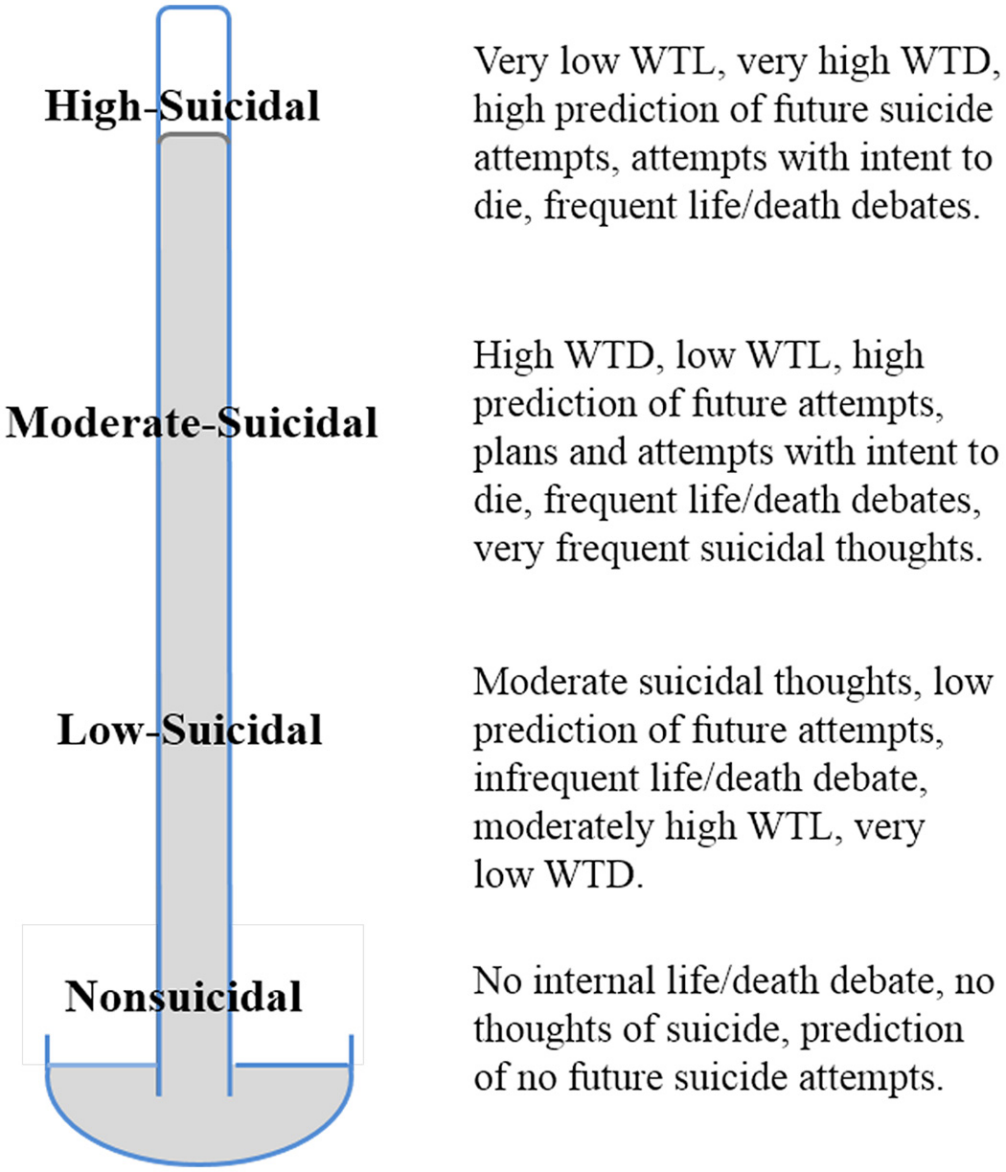
The suicidal barometer model with descriptions of suicidality levels based on item response theory analyses. WTL = wish to live, WTD = wish to die.

These findings provide good rationale for considering the relevance of Eagly and Chaiken’s [9] tripartite model to suicide. The SABCS also relates well to suicide-specific theory. WTL and WTD capture an affective characteristic of suicidality, and also what many theorists regard as a key communality of suicidal minds, i.e., life/death ambivalence [23, 41, 111]. The Debate item then captures an intense cognitive aspect of the internal life/death struggle [103]. The importance of these items to the scale provides support for suicide theories and assessment emphasizing the lived experience of being suicidal. The value of the Ideation item validates including a general item on suicidal cognition. The importance of the Prediction item helps validate the individual’s ability to assess their own suicide risk [112]. The Behaviors item functioned best at discriminating low to moderate levels of suicidality, and is useful for understanding the individual’s suicidal background. However, consistent with previous findings, communication of suicidality lacked validity for suicide risk assessment [36, 37]. The suicidal barometer model encompasses current personal distress and prediction of future distress and behaviors. Fig 2 illustrates how SABCS items, through IRT analyses, can contribute to a more precise model.

### Future SABCS Development

There were some important limitations to this study. Notably, a larger and more heterogeneous sample is required to better validate the predictive abilities of the SABCS. In addition, although online assessment has some advantages over face-to-face, pen-and-paper, and telephone interviewing, a random sample of a known population is needed to demonstrate population prevalances of the individual item attributes. This study substantiated the benefits of using IRT analyses for testing item and scale attributes [48, 53, 113]. The only known study to previously use IRT to develop a suicide-related test was on knowledge of suicide postvention [113]. Nader et al. found IRT, and other rigorous psychometric testing, useful for producing a more robust measure. The SABCS was tested on high-risk clinical, university student, and three community samples. Items were tested with different response ranges and for various timeframes (e.g., lifetime, past two weeks). Those results showed very similar psychometric properties, indicating that the different response ranges and timeframes had little effect on reliability or validity, as assessed by traditional CTT analyses. However, IRT analyses revealed that more than 6–7 response categories is not effective and should be avoided for these items. It was also tested through pen-and-paper, computer-administered, and online modes. Surveys, however, do not allow for follow-up questions, verification of responses, or other important assessment techniques. Similar to Hatcher and Pimentel’s study [114], it would be very useful to test the SABCS through clinical face-to-face interviewing in comparison with self-report methods, to determine any effects of social desirability bias, faking, or other sources of assessment error. The SABCS demonstrated robust properties throughout these variations. However, further analyses are required to test validity with adolescents, other ethnicities, and in other languages. The Behaviors item preformed less well than others and might benefit from further development. Based on the present findings, we recommend 6–7 level response formats, with slightly higher weighting of WTD (see Appendix), but ideal weighting remains undetermined. Recent study has shown that suicidal typologies are useful for understanding current and future risk [112]. Further analyses are also required to determine valid cut-off scores for similar risk groupings. Lastly, as a public domain instrument, further experimentation and development are encouraged.

### Conclusions

This study examined theoretical and empirical interpretations of suicidality and found robust evidence that affective, behavioral, and cognitive aspects of the life-death struggle are useful for valid evaluations. Through systematic analyses, employing IRT and CTT methodologies, and attending to the minutiae of scale infrastructure, the resulting Suicidal ABC Scale demonstrated incremental improvements over an existing standard. It improves on past measures by including all ABC attributes, allowing clinicians some insight into the experience of the suicidal mind. The SABCS is a brief, public domain, reliable and valid measure of suicidality/suicide risk. It is appropriate for public screening, research, and clinical purposes, including the assessment of clinically meaningful changes in suicidality.

## Appendix

### The Suicidal Affect-Behavior-Cognition Scale (SABCS)

#### Instructions to test administrators

Present the scale as shown below, but without the item response scores. Administration is ideally done anonymously and in non-threatening environments. Note that time frames, italicized, may be altered.

We would like to ask you some personal questions related to killing oneself. Please indicate the response that best applies to you.

1. **Have you *ever* thought about or attempted to kill yourself?**
  - Never (0)
  - It was just a brief passing thought (1)
  - I have had a plan at least once to kill myself but did not try to do it (2)
  - I have attempted to kill myself, but did not want to die (3)
  - I have had a plan at least once to kill myself and really wanted to die (4)
  - I have attempted to kill myself, and really wanted to die (5)
2. **How often have you thought about killing yourself in the past *year*?**
  - Never = (0) (1) (2) (3) (4) (5) = Very Often
3. **In the past *year*, have you had an internal debate/argument (in your head) about whether to live or die?**
  - Never = (0) (1) (2) (3) (4) (5) = Frequently
4. ***Right now*, how much do you wish to live?**
  - Not at All = (5) (4) (3) (2) (1) (0) = Very Much
5. ***Right now*, how much do you wish to die?**
  - Not at All = (0) (2) (3) (4) (5) (6) (7) = Very Much
6. **How likely is it that you will attempt suicide someday?**
  - Not at All = (0) (1) (2) (3) (4) (5) = Very Likely

## Supporting Information

S1 Dataset (TXT)
