# Correction: The ABC’s of Suicide Risk Assessment: Applying a Tripartite Approach to Individual Evaluations

**DOI:** 10.1371/journal.pone.0133223

**Published:** 2015-07-14

**Authors:** The PLOS ONE Staff

The affiliation for the fifth author is not correct. Christopher H. Willcox is not affiliated with #6 but with #3 School of Psychology, University of Newcastle, Newcastle, NSW, Australia. The publisher apologizes for the error.

The Corresponding Author email is not correct. The correct email address is: KHarris@psy.uq.edu.
